# Widespread use of National Academies consensus reports by the American public

**DOI:** 10.1073/pnas.2107760119

**Published:** 2022-02-22

**Authors:** Diana Hicks, Matteo Zullo, Ameet Doshi, Omar I. Asensio

**Affiliations:** ^a^School of Public Policy, Georgia Institute of Technology, Atlanta, GA 30332;; ^b^Andrew Young School of Policy Studies, Georgia State University, Atlanta, GA 30303;; ^c^Princeton University Library, Princeton University, Princeton, NJ 08544;; ^d^Institute for Data Engineering & Science, Georgia Institute of Technology, Atlanta, GA 30308

**Keywords:** BERT, natural language processing, public understanding of science, machine learning

## Abstract

In seeking to understand how to protect the public information sphere from corruption, researchers understandably focus on dysfunction. However, parts of the public information ecosystem function very well, and understanding this as well will help in protecting and developing existing strengths. Here, we address this gap, focusing on public engagement with high-quality science-based information, consensus reports of the National Academies of Science, Engineering, and Medicine (NASEM). Attending to public use is important to justify public investment in producing and making freely available high-quality, scientifically based reports. We deploy Bidirectional Encoder Representations from Transformers (BERT), a high-performing, supervised machine learning model, to classify 1.6 million comments left by US downloaders of National Academies reports responding to a prompt asking how they intended to use the report. The results provide detailed, nationwide evidence of how the public uses open access scientifically based information. We find half of reported use to be academic—research, teaching, or studying. The other half reveals adults across the country seeking the highest-quality information to improve how they do their job, to help family members, to satisfy their curiosity, and to learn. Our results establish the existence of demand for high-quality information by the public and that such knowledge is widely deployed to improve provision of services. Knowing the importance of such information, policy makers can be encouraged to protect it.

## Significance

Advocates for open access argue that people need scientific information, although they lack evidence for this. Using Google’s recently developed deep learning natural language processing model, which offers unrivalled comprehension of subtle differences in meaning, 1.6 million people downloading National Academies reports were classified, not just into broad categories such as researchers and teachers but also precisely delineated small groups such as hospital chaplains, veterans, and science fiction authors. The results reveal adults motivated to seek out the most credible sources, engage with challenging material, use it to improve the services they provide, and learn more about the world they live in. The picture contrasts starkly with the dominant narrative of a misinformed and manipulated public targeted by social media.

The quality of information in the public sphere and its dissemination are of increasing concern, with scholars examining information bubbles, fake news, the spread of misinformation, manipulation of social media users, Twitter bots, etc. This growing research corpus seeks to understand the corruption of the information ecosystem. Although important, this focus on dysfunction obscures the fact that large parts of the public information sphere continue to function very well. However, lacking a crisis narrative, the high-performing aspects of the system often escape attention. Without systematic research into the information ecosystem’s healthy facets, our capacity to protect and develop existing strengths will be limited. This paper addresses this gap, focusing on use of high-quality information in the public sphere—research-based, consensus reports produced by the National Academies of Sciences, Engineering, and Medicine (NASEM)—and their use outside academe.

NASEM reports are written by committees of scientific experts and represent their consensus on the state of knowledge about a topic. NASEM’s voice is highly credible, a characteristic NASEM has carefully nurtured and maintained, transcending even the internet-driven “death of expertise” (1). That NASEM reports are used by the federal government agencies that often commission them and by professors in their research and teaching would seem obvious. But, beyond that, does anyone use the reports?

In 2011, NASEM made their reports free to download, that is, open access. Accessibility does not equate to use, of course. Therefore, we must ask, do nonscientists, or laypersons, who are not steeped in the foundational knowledge required to understand state-of-the-art technical publications, use this information? If so, why do they seek it out, and what needs does it satisfy? Answering these questions will provide insight into the functioning of a high-quality information ecosystem in a technologically challenging age. When downloading reports, users are asked to “please take a moment and tell us how you will be using this PDF.” Here we provide analysis of the pattern of 6.6 million downloads since 2003 and the 1.6 million comments left since 2011 in response to this prompt by those with a US IP address. We provide empirical insight into public use of National Academies reports, and, more broadly, the use of open access science.

## Materials and Methods

Table 1 provides a basic description of the dataset: number of reports, downloads, and comments. About half of downloads and 64% of comments were from US IP addresses, and these were the focus of this analysis. Overall, the number of comments was 22% of the number of downloads. That figure was 12% in the early years, 29% in 2016, and 23% in other years. The share of downloaders that left a comment was actually higher because, often, people downloaded more than one report but left a comment only on the first one. The most frequent comment was simply “research,” which accounted for 7.5% of comments.

**Table 1.** Description of NASEM dataset

	Number or year	Date or percentage
Reports downloaded after 2002 in the United States	10,275	
First download	2003	June
First comment	2011	June
Last download and comment	2020	February 6
Worldwide downloads	16,000,616	
US downloads—raw data	8,303,511	52%
US downloads—processed*	6,648,781	
Worldwide comments	2,433,199	
US comments^†^	1,554,157	64%
Most frequent US comment—“research”	116,828	7.5%
Unique US comments	862,258	

*Downloads from Chinese domains that appeared under a US IP address were removed, as were duplicates, algorithmic downloads, and multiple copies of the same report downloaded by a user in 1 d.

^†^Excluded from the analysis were 2,051 comments classified as refusal to answer the prompt. Examples included ppoo00, nan, kjbkbknln, and similar.

In addition to the IP address, the data provide the domain of the users’ email address (i.e., @gatech.edu). Classification of domains reveals that users of NASEM reports were varied; see Table 2. Universities accounted for 29% of US downloads, and companies accounted for 11%. Gmail and internet service provider (ISP) email accounts represented 35% of downloads, indicating both a great deal of personal use and many people using Gmail accounts for work purposes (2).

**Table 2.** US downloads of NASEM reports and comments by sector

Sector	Number				Percentage			
	Downloads	Users	Domains	Comments	Downloads	Users	Domains	Comments
Gmail & ISP	2,300,947	926,227	22,796	491,003	35	38	11	32
University	1,903,312	699,854	16,543	451,816	29	28	8	29
Companies (.com)	719,160	274,546	97,542	171,163	11	11	45	11
Federal Government	450,798	121,830	2,333	91,587	7	5	1	6
Nonprofit (.org)	331,821	124,390	38,671	92,245	5	5	18	6
State & Local Government	223,044	72,217	8,497	67,824	3	3	4	4
School	205,089	102,148	14,069	65,627	3	4	7	4
Health Care	168,637	69,046	3,765	56,897	3	3	2	4
Consulting	96,985	23,240	1,326	23,007	1.5	0.9	0.6	1.5
NASEM	85,602	5,889	1,732	3,491	1.3	0.2	0.8	0.2
Transportation	78,703	14,481	381	19,649	1.2	0.6	0.2	1.3
Miscellaneous (.net etc.)	55,514	18,871	7,194	12,847	0.8	0.8	3.3	0.8
Media	9,604	3,768	473	2,303	0.1	0.2	0.2	0.1
Museum	9,142	2,904	412	2,745	0.1	0.1	0.2	0.2
Community College	9,121	4,856	354	1,955	0.1	0.2	0.2	0.1
Total	6,647,479	2,464,267	216,088	1,554,159	100	100	100	100

Table 3 lists the titles, publication years, and approximate download counts for the 25 most downloaded reports. The most downloaded report, *A Framework for K-12 Science Education*, forms the basis for the Next Generation Science Standards (NGSS), which were broadly adopted guidelines for teaching science in schools (3). The second most downloaded report, *The Future of Nursing*, was part of a movement that led to a campaign launched by AARP and the Robert Wood Johnson Foundation to advance the nursing profession (4). The report’s recommendations included removing scope-of-practice barriers and, by 2020, aiming to have 80% of nurses with a bachelor’s degree (BSN) and double the number of nurses with a doctorate. Shortly before these two landmark reports were released, the National Academies Press removed the paywall on its reports. Thus, those without access to a research library, such as schoolteachers and nurses, were able to download the NGSS and nursing reports free of charge from the day of their launch.

**Table 3.** Most downloaded reports

Downloads (thousands)	Pub year	Title
206	2012	*A Framework for K-12 Science Education Practices, Crosscutting Concepts, and Core Ideas*
125	2011	*The Future of Nursing: Leading Change, Advancing Health*
74	2000	*How People Learn: Brain, Mind, Experience, and School: Expanded Edition*
59	2009	*On Being a Scientist: A Guide to Responsible Conduct in Research: Third Edition*
45	2001	*Crossing the Quality Chasm: A New Health System for the 21st Century*
43	1996	*National Science Education Standards*
38	2017	*The Health Effects of Cannabis and Cannabinoids*
36	2000	*To Err Is Human: Building a Safer Health System*
35	2008	*Science, Evolution, and Creationism*
33	2008	*Ready, Set, SCIENCE! Putting Research to Work in K-8 Science Classrooms*
28	2007	*Rising Above the Gathering Storm*
26	2013	*Best Care at Lower Cost: The Path to Continuously Learning Health Care in America*
26	2015	*Dying in America: Improving Quality and Honoring Individual Preferences Near the End of Life*
22	2010	*A Data-Based Assessment of Research-Doctorate Programs in the United States*
21	2016	*Genetically Engineered Crops: Experiences and Prospects*
21	2001	*Adding It Up: Helping Children Learn Mathematics*
20	2018	*How People Learn II: Learners, Contexts, and Cultures*
20	2015	*Guide to Implementing the NGSS*
20	2014	*The Growth of Incarceration in the United States: Exploring Causes and Consequences*
20	2013	*Priorities for Research to Reduce the Threat of Firearm-Related Violence*
20	2013	*US. Health in International Perspective: Shorter Lives, Poorer Health*
18	2014	*Developing Assessments for the NGSS*
18	2011	*Successful K-12 STEM Education*
17	2010	*Rising Above the Gathering Storm, Revisited: Rapidly Approaching Category 5*
17	2011	*Relieving Pain in America: A Blueprint for Transforming Prevention, Care, Education, and Research*

Top 25 most downloaded NASEM reports out of a total of 10,275 reports. Downloads by US based IP addresses only. Downloads counted June 2003-February 2020.

To explore the use of NASEM reports, we classified the 1.6 million comments into 64 use categories, applying transformer neural networks, a recent class of pretrained contextual language models, to accurately detect long-tail discussion topics with imbalanced data. This capability has been elusive with prior approaches. Specifically, comments were classified using Bidirectional Encoder Representations from Transformers (BERT), a transformer-based natural language machine learning classification algorithm with outstanding performance on subtle classification tasks because it encodes both semantics and the rich latent structure of sentences (5, 6). The superiority of BERT over other machine learning natural language classification models has been repeatedly established in varied real-world social science datasets (7–12).

The 64-category taxonomy of report use, and the ground truth dataset used to train the model, were created in two stages. First, a pilot study was conducted using keyword searching to classify comments and build the taxonomy. Second, pilot categories were random sampled, and each comment in the sample was inspected by two independent readers. For a comment to enter the ground truth set, the two readers agreed that only one category applied and further agreed on the category. The BERT model was fine-tuned by training on the ground truth set, and then the fine-tuned model was used to predict categories for all 1.6 million comments. The comment text had been cleaned to prepare for algorithmic processing. Each comment was classified into one category only, which is a simplification, as comments sometimes do report multiple intended uses, a common example being “education and research.” The method is fully described in *SI Appendix*, and the data-cleaning python and BERT notebooks, model output weights, and appendix table containing category details as well as keywords used to construct pilot classification are in Figshare: https://figshare.com/articles/dataset/BERT_Model_weights_for_Widespread_use_of_National_Academies_consensus_reports_by_the_American_public_/14605839/3. Data are proprietary to the National Academies Press and must be requested from them.

Our study has limitations. We only examined US comments and therefore cannot say how reports are used in other countries, although the data indicate that reports are used around the world. Strictly speaking, our discussion provides insight into the 22% of downloads that were commented, although generalization to all downloaders appears warranted, as commenters appear to be representative of the population of downloaders; see *SI Appendix*. We were not able to implement multilabel classification, applying more than one category to a comment, although comments sometimes do mention more than one use. This would be a topic for further research with more specialized semantic annotation. Our insights are limited by what commenters divulge, by their withholding of detail, and by ambiguity in phrasing. Furthermore, commenting is contemporaneous with downloading, and, in many cases, use of a report may not be anticipated but may develop over the months following download. Although this might be captured if downloaders were asked several months after download how they used a report, such a survey would likely not get responses from those whose use was transient or who decided not to use the report, and therefore such a survey would miss information we capture here.

**Fig. 1.**
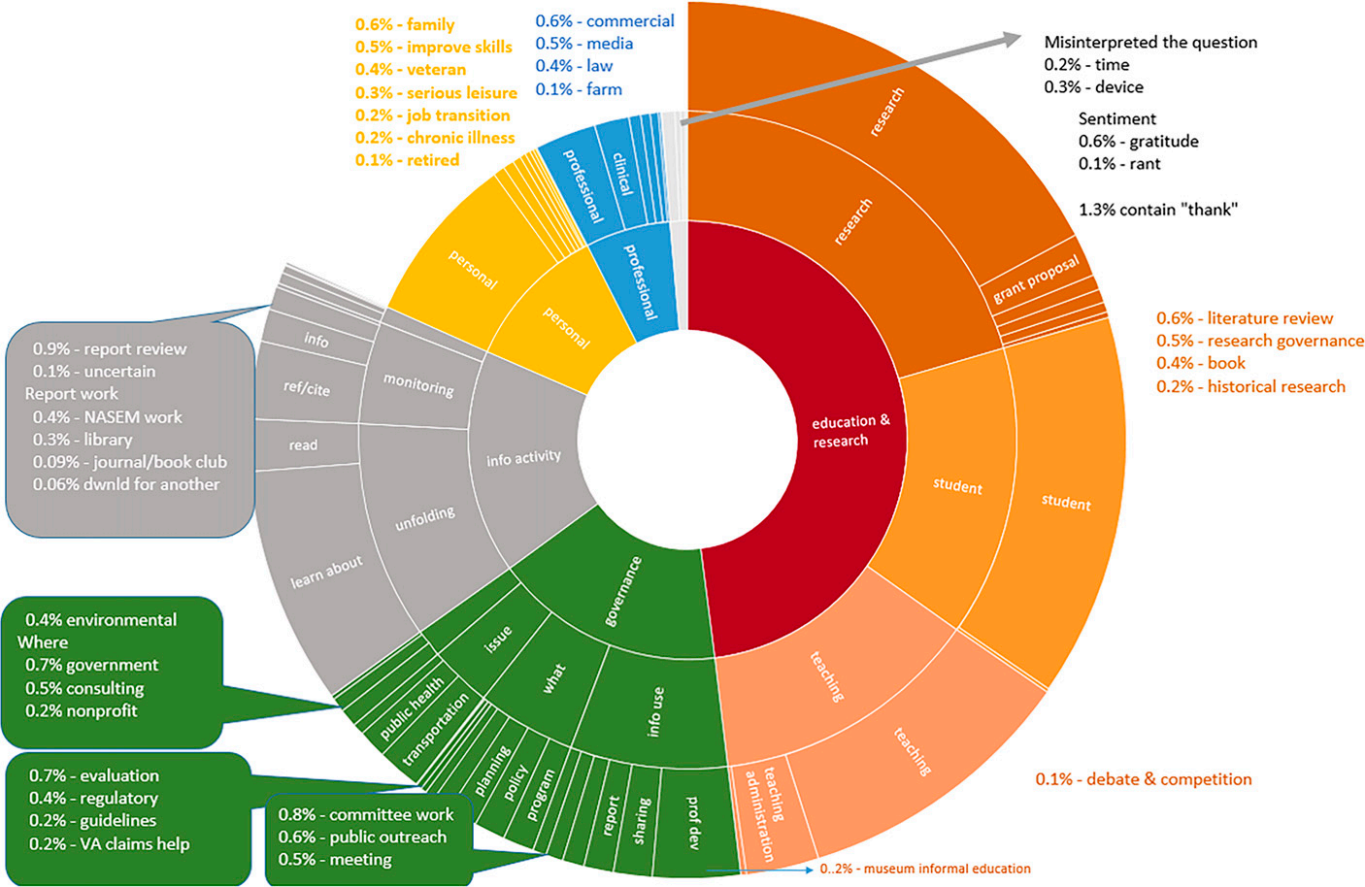
How  were NASEM reports used? Classification into 64 categories of 1.6 million comments left by US downloaders of NASEM reports between 2011 and 2020. Downloaders were asked how they will use the report. BERT machine learning algorithm was used to classify.

Fig. 1 summarizes the category structure and results in graphical form. Note that 1% of comments equals 15,000 comments. *SI Appendix*, Table S2 reports statistics for each category, and a table providing detailed information on each category is available here: https://figshare.com/articles/dataset/BERT_Model_weights_for_Widespread_use_of_National_Academies_consensus_reports_by_the_American_public_/14605839/3. Two statistics are referenced in the discussion. Correlation with the overall distribution refers to the correlation coefficient between the category and overall distributions of comments over reports. Concentration refers to the Herfindahl Index across reports, a measure of how comments are concentrated on a few reports (higher number) or spread evenly across many reports (a lower number).

## Results

### Academic and Miscellaneous Report Use.

Scholarly users engaged in research, teaching, and student studying accounted for half of report use (48% of classified US comments). This share is higher than the 33% of comments associated with a school or university domain, because many downloaders provided a Gmail or ISP email address (2). Research accounted for 20% of report use, students studying for classes accounted for 15%, teachers using reports to prepare courses accounted for 10%, and school administration, for example, curriculum development, accounted for 2.7%. Subcomponents of these categories, each accounting for less than 1% of comments, included museum informal education (in teaching), debate and competition (students preparing for competitions like the National Ocean Sciences Bowl), and, in research, the following: literature review, historical research, writing books, and research governance (institutional review boards, institutional animal care and use committees, and laboratory safety or animal care). Fig. 1 plots the full results.

The pattern of report reading in academia largely correlates with the overall pattern, because scholarly use accounts for such a large share of overall usage. The correlation with the overall distribution of downloads across reports was high—teachers, 0.85; research, 0.80; and students, 0.77. Teachers most often downloaded the NGSS report (14% of teacher comments) and accounted for 36% of the comments associated with that report. Added to this were teaching administrators, developing curriculum for schools or districts, for example. They accounted for 2.7% of all comments but 17% of comments on the NGSS report. In total, teachers, teaching administrators, teaching coaches (professional development in schools), and student teachers account for 72% of the NGSS report users. Other reports downloaded by teachers included *How People Learn* or concerned the implementation of NGSS and STEM teaching. Given the strong emphasis in the *Future of Nursing* report on university education, it is not surprising that 61% of this report’s comments report use by students, often studying for BSN degrees, and 11% of student comments were associated with this report. The NGSS report is the fourth most common student download, because people studying for education degrees use it. The downloading pattern for research purposes was slightly less correlated with the overall pattern, because the Nursing and NGSS reports were the first and third most downloaded reports. The second most downloaded report for research purposes was *The Health Effects of Cannabis*, accounting for only 0.8% of research downloads. No single report accounted for more than 1% of research downloads, suggesting that research interest is spread evenly across reports. Thus, the Herfindahl index for concentration across reports is very low in the research category; see *SI Appendix*, Table S3.

Nineteen thousand comments did not describe report use and were classified as “other.” Almost 6,400 of these comments expressed gratitude. This type of comment was more common in the early years when free access was novel. No particular report was favored by those expressing gratitude. Another 2,800 comments provided opinions on issues but no indication of how the report would be used, that is, were rants. Ranters’ concerns included gun violence, evolution and creationism, genetically engineered crops, and cannabis. Seven thousand seven hundred comments involved misreading of the prompt by people who reported how long they expect to use the report (hours, days, months, etc.) or the device they read the report on (laptop, iPad, etc.). The distribution of these comments across reports was highly correlated with the overall distribution (0.87 and 0.96), indicating misreadings were random with respect to report contents.

Academic use of NASEM reports is expected, and we are not surprised to learn that teachers were very interested in science standards promulgated in NASEM reports. Some misinterpretation of the prompt was also inevitable and of little interest. The mystery is, Why are scientifically grounded reports, largely commissioned by federal agencies to meet their needs, of any interest to the general public? The taxonomy we develop below answers this question in detail, uncovering the wide variety of uses citizens make of high-quality scientific information. The 52% of US comments outside the scholarly and other categories fell into three broad areas: everyday information activity (17% of comments), governance work (18%), and self-directed learning, both professional (6%) and personal (10%); see Table 4. The activities are heterogeneous and cannot be encompassed by one theoretical framework. Of necessity, therefore, we are eclectic in our interpretation drawing on ideas of everyday information use, new public governance, and self-directed learning (13–16).

**Table 4.** Six broad categories of NASEM report use

Category	Comments	Share, %	Accuracy	F1 macro
Education and research	752,985	48	0.91 (0.006)	0.91 (0.006)
Governance	279,799	18	0.93 (0.006)	0.92 (0.004)
Information activity	262,209	17	0.80 (0.012)	0.82 (0.006)
Personal	157,144	10	0.87 (0.009)	0.89 (0.008)
Professional	86,854	6	0.83 (0.012)	0.84 (0.010)
Other	15,168	1	0.87 (0.017)	0.86 (0.017)
Overall	1,554,159	100	0.89 (0.004)	0.87 (0.004)

Broad classification into six categories of 1.6 million comments left by US downloaders of NASEM reports between 2011 and 2020. Downloaders were asked how they will use the report. BERT machine learning algorithm was used to classify. The accuracy, F1 macro, and SEs (reported in parentheses) were generated by running 10-fold cross-validation of the optimized model. Accuracy and F1 macro are the average over the 10 runs.

### Everyday Information Activity.

The first broad category—information activity—involves the report as opposed to life activities focused on work or leisure goals. Information activity categories were required for comments that do not mention, the more interesting to us, life activities. Table 5 organizes the information activity categories using a taxonomy of everyday information use developed by Hektor (13). For example, a handful of people were downloading for their boss or, less often, a neighbor or friend. About twice as many people were uncertain how they intended to use the report, marking them as people browsing for information that they believe might prove useful, partly depending on the report’s contents. This small group of people, 0.20%, provided insight only into their first stage of information use, seeking out the report.

**Table 5.** Taxonomy of everyday information use and associated comment categories

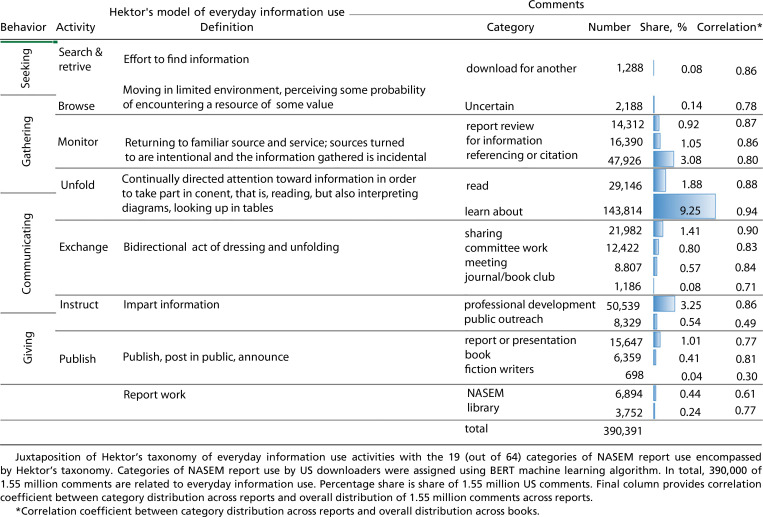

Somewhat more people volunteered that they were gathering information from the report. Report review included editors fact-checking other documents that referenced the report as well as those taking a somewhat skeptical stance to the report and reviewing its recommendations. The category “for information,” accounting for 1% of comments, refers to those gathering background information without stating a purpose. Referencing or citation checking refers to people gathering reference material or using the report for general reference. After NGSS and nursing, downloads here included handling chemical hazards in laboratories, care of laboratory animals, roundabouts, and dietary reference intakes. Monitoring, meaning intentionally turning to a familiar source, seems to encompass these uses. Monitoring accounted for 5% of comments.

Hektor applied the term “unfold” to the activity of directing attention toward information in order to take part in its contents. We applied this concept to those who said they were going to read the report (2%) and those whose purpose was to learn about the report’s topic (9%). Note that we did not use these information categories if a comment also mentioned a life activity. In such cases, we classified the comment into that life activity. In other words, these categories are residual, used when there was no alternative. Comments in these categories were distributed across reports randomly, indicated by a high correlation between the distribution of comments across reports in these categories and the overall distribution; see *SI Appendix*, Table S3. This suggests the categories convey minimal insight.

Exchanging information is the next, more involved information activity. In the NASEM data, four categories expressed the concept of interacting with others about the report. Sharing or discussing the report, most often with colleagues, accounted for 1.4% of use. Particular to this category was the fourth most shared report, the 2018 examination of sexual harassment of women in academia. Six percent of users of this report intended to share it. Preparing for task forces, advisory committees, or working groups represented 0.6% of report use. The sexual harassment report was the fourth most common in this category. Sharing reports and committee work are two life activities that are also part of governance, discussed below. Preparing for workshops and conferences accounted for another 0.5% of comments. Finally, journal and book clubs read NASEM reports; over 1,300 comments referred to this activity, representing both work and leisure activity. Book clubs read *How People Learn*, as well as many reports on STEM teaching.

Hektor’s instructing activity encompasses both professional development (3%) and public outreach (0.5%). Professional development involved, among other things, instructional coaches in school, certification examinations, and interprofessional education for health care providers. Most outreach was STEM outreach, high school programs, or community engagement work, and the most downloaded report was *Communicating Science Effectively*. Hektor’s final information activity is publish. In this activity, we find those using the report to prepare their own white paper, talk, or conference presentation (1%). These people most often downloaded health care reports. Also in “publish” were authors of books. About 6,400 of those conducting research mentioned it was for a book. Nonfiction authors were interested in a broad range of reports, most often concerned with incarceration, dying, NGSS, and the poor state of health in this country. Authors of fiction, most often science fiction, as well as a few visual artists appeared in the NASEM comments 700 times. They were most interested in understanding severe space weather events, forensic science, and reflecting sunlight to cool the planet.

Two final categories seemed to be information activities that did not fit into Hektor’s framework because they were not everyday use. Authors of NASEM reports, members of report committees, and NASEM staff who worked on reports accounted for about 6,900 comments. Librarians adding reports to their libraries accounted for 4,000 comments. Their third most downloaded report was *Preparing the Workforce for Digital Curation*.

### Use in Governance.

Governance accounted for 17% of comments. Theoretically, the concept of new public governance directs our attention to the network of government, nonprofit, and civic organizations engaged in the collaborative solving of societal problems (14). For societal problems addressed in NASEM reports, the comments provide a window into the activities of this network of actors across a range of substantive issues. However, there is significant variation in how activities were described, with similar activities likely described differently by different people. At one extreme, some comments say just “government work.” At the other, a person provided the name of the nonprofit they worked for and/or the interorganizational committee they were preparing to present to and described the purpose of the committee’s strategic planning activity. Therefore, categories cannot be mutually exclusive or have clear boundaries. Having said that, report downloaders work in consulting (0.5%), government agencies (0.7%), and nonprofits (0.2%) and work on issues in transportation (Transportation Research Board reports) (1.7%), public health (1%), and environment (0.4%). They were planning and developing strategies, designing and managing programs, evaluating, developing guidelines and regulations, accrediting, helping veterans with disability claims, and working with legislators to develop policy, all of which accounted for 5% of the comments. Some categories discussed earlier as information activities also are governance activities—professional development, committee work, sharing with colleagues, preparing for meetings, and writing reports or presentations.

### Self-Directed Learning.

Comments about applying knowledge in professional or personal life were notable for the sense of self-directed learning (15) they conveyed. The picture that emerges is of adults outside of any formal structure, even professional development courses, seeking to improve their job skills, to improve the quality of the clinical or pastoral care they provide, to improve the diet of their animals, or to improve the grounding of the universes they imagine in their science fiction novels. Others in the media sought to provide pointers to these self-education resources to the interested public.

Work-related use, with little to no further specification, accounted for 3% of commenting and was correlated with overall commenting (0.74). Often, however, additional information was provided. Doctors and nurses intending to apply the knowledge gained to their clinical work left 1.7% of comments and were most concerned with dying, cannabis, and improving diagnosis. A purpose associated with a firm, such as “for my business” or “market research,” amounted to 0.7% of comments (although 173,000 comments were associated with a .com domain). Beyond cannabis and NGSS, there was interest in *Best Care at Lower Cost*, genetically engineered crops, and cost–benefit of nonemergency medical transportation. Journalists writing about the report in an article or others mentioning it in websites and newsletters left 0.6% of comments. They were most concerned with genetically engineered crops, immigration, cannabis, and incarceration. Lawyers left 0.4% of comments when downloading NASEM reports such as *Assessing Eyewitness Identification*, *Reference Manual on Scientific Evidence,* and *Strengthening Forensic Science*. Farmers interested in reports such as *Nutrient Requirements of Beef Cattle* accounted for 0.1% of comments. Finally, pastors and chaplains, often working in hospitals, accounted for about 400 comments, downloading, for example, *Dying in America* and *The Growth of Incarceration*. Including the fiction writers mentioned earlier, in total, 6% of comments related to these professional uses.

Personal use accounted for 10% of comments, 8% being undifferentiated personal use concerned with cannabis, dying, genetically engineered crops, evolution versus creationism, and reducing gun violence. When people provided more detail, they were often helping family members (0.7%), most often helping children with math. Others were veterans working on a VA disability claim (0.4%) and using some of NASEM’s 20 reports on Agent Orange’s effects or other reports on the health effects of burn pits or high noise levels. Serious leisure activity accounted for about 4,300 comments. Serious leisure is a concept articulated by Stebbins (16) that describes unpaid activities engaged in by adults over a long time and often involving self-directed learning. Volunteer work, blogging, ham radio, and amateur astronomy enthusiasts used NASEM reports on, for example, frequency allocation, lost crops of the Incas, or astronomy, to pursue their interests. Improving skills is another well-known motivator for adult learning (15) that accounted for about 6,300 comments. The improving skills category holds comments like “personal professional development” that appeared to be self-directed learning not associated with a formal course (which would be classified as professional development). Retired people left at least 2,300 comments, sometimes divulging their age, often expressing a personal interest in keeping up with areas related to their former profession. Most downloaded reports concerned dying, cognitive aging, NGSS, and preventing dementia. Job transitions are another prompt to self-directed learning recognized in the literature (15). Over 4,400 comments mentioned preparation for job interviews, exploring career changes, or becoming familiar with a new job area. People dealing with illness and interested in the NASEM reports on chronic fatigue syndrome, pain, and cannabis left over 2,600 comments.

## Discussion

While *SI Appendix*, Table S3 reports results in detail, the three-layer sunburst chart in Fig. 1 summarizes the results described above. It displays our taxonomy of the comments left by downloaders of NASEM reports in response to the prompt “How will you use this report?” The taxonomy identifies detailed use categories and groups them in relation to each other and to theoretical frameworks.

The 765,000 people who indicated their reasons for wanting a report provide rich insight into the public’s need for high-quality, scientifically based information. Their distribution across states and sectors (Table 2) is the same as the full set of downloaders (*SI Appendix*), suggesting that our conclusions can be generalized to all downloaders of NASEM reports between 2011 and 2020. These data provided a uniquely large-scale picture of an informal activity known as self-directed learning or lifelong learning (15). Because it is easier to see, lifelong learning is often studied in formal educational settings (17, 18), and we saw that here in the use of reports by providers of continuing education and professional development programs. This was in addition to the extensive report use by professors planning university courses or students using reports in coursework or theses.

The demographic characteristics of the commenters are unknown. However, they are self-directed adult learners, and those who engage in self-directed, adult learning are known to be higher income, in professional occupations, and better educated than nonparticipants (15). The nature of the comment text suggests authorship by educated professionals and is notably different from text in tweets, for example. We see hospital chaplains, science fiction authors, and lawyers striving to improve the practice of their chosen profession. Doctors sought to improve their clinical practice, nurses sought to improve the institution of nursing, and teachers sought to raise the standards of science teaching in our schools. Many professionals used reports to keep up with developments in their profession through personal professional development, outside of requirements or formal processes.

Similarly uplifting is the view of governance provided by downloaders working in government agencies, nonprofits, and consulting firms, often sharing reports with each other, seeking to use the highest-quality information available to guide their work, pursued in committees, working groups, workshops, and presentations as, together, they work through complicated public issues. People also perused NASEM reports for personal edification. Excluding one NASEM report addict who downloaded 551 times for “personal edification” over almost the entire time span, 3,700 comments mention edification. Outside of scholarly use, the largest groups of users were the 91,000 people who downloaded more than 130,000 reports simply to learn more about the report’s topic, and the 70,000 people using 129,000 reports for personal reasons.

These insights align with other analyses of public use of scholarly information. We find that about half of report use does not involve studying, teaching, or research. After Medline was made open access in 1997 and use exploded, the National Library of Medicine estimated that one-third of users were members of the public, broadly defined (19). Alperin (20) examined the use of open access science platforms in Latin America and estimated that about one-quarter of use came from outside academia. The British Library’s document request pattern in 2010 suggested that 45% of requests came from professionals, businesses, or individuals outside academia (21).

Patient interest in trustworthy medical information on the internet was recognized early and has been thoroughly explored (22, 23). We found that people suffering from illnesses seek out NASEM reports, as do their family members (24). Empirical investigations of the reasons behind nonmedical public use of open access scientific information are just beginning. In November 2020, SpringerNature released a report focused on the use of open access journal articles. It included empirical examination of use of open access science, surveying 6,000 downloaders of research articles, asking, among other things, how they intended to use the document. Their results broadly align with ours, finding high levels of academic use, lots of learning, sharing, writing other documents or presentations, planning further research, and what they describe as “a long-tail of miscellaneous reasons” (25)—which we are able to probe in detail here.

Also related are the information ecosystems in professions which include conferences, professional magazines, and professional social media (26, 27). Formal professional education includes professional development courses and continuing education related to licensing requirements which appear here as providers using NASEM reports in developing their instructional material. Curiosity motivated many of the adults who download NASEM reports, and, in governance, a case has been made for the central role curiosity could play as an organizing principle for public management (28).

As for informal sources of information for motivated adults, public libraries serve a role similar to NASEM (29). Surveys probing use of public libraries ask about similar types of use: as a student, improving job skills, executing work tasks, professional development, health issues, reading nonfiction as a leisure activity, interest in history or society, and participating in societal discussions (30). Although the physical presence of a library building creates some divergence, work on the value of libraries surfaces similar motivations for public information seeking as found here. A review by Stenstrom et al. (31) identified “three umbrella categories of social value with notable facets” that we also see in NASEM report use. First is support for personal advancement, both personal learning and learning related to finding a job or increasing job skills. Second is support for vulnerable populations, which we see in the use of NASEM reports by veterans submitting VA disability claims and those who help them with their claims. They rely heavily on the series of NASEM reports on Agent Orange, burn pits, and other issues. Third is community development, under which is support for generalized trust, fostered by NASEM’s maintenance of a high level of credibility. Community development is also supported by NASEM reports on disaster response and community resilience. Before the pandemic, NASEM had published over 100 reports with disaster, crisis, or resilience in the title. These reports gathered 26,000 comments from people who are often making state or local disaster response plans.

The overall impression is of adults motivated to reach higher, seek out the most credible sources, engage with challenging material, and use it to improve the services they provide or learn more about the world they live in. It is a heartwarming picture that expands the standard public information narrative referencing only social media. The widespread interest in NASEM reports by adults in this country justifies the public money spent to produce the reports and validates the decision to make them accessible through free download. Open access to NASEM reports provides a hitherto unrecognized benefit to the public who have put the reports to many and varied uses over the decade since they became available. Assisted by media reporting on newly published reports, people seek them out and download them because they need or are curious about the high-quality information contained within. Probably, most Americans have benefited from NASEM report readers’ work, whether through the improved science education their children received, the new roundabout on their commute designed with guidance from NASEM reports, nursing care received during a hospital stay, or the science fiction book they just finished reading. Many aspects of our lives have been improved by information provided in National Academies’ reports. Our study reveals strong demand for the highest-quality information in the public sphere and hints at how such information has improved the services our public and health care sectors provide.

## Supplementary Material

Supplementary File

## Data Availability

The data-cleaning python and BERT notebooks, model output weights, and appendix table containing category details as well as keywords used to construct pilot classification are in Figshare: https://figshare.com/articles/dataset/BERT_Model_weights_for_Widespread_use_of_National_Academies_consensus_reports_by_the_American_public_/14605839/3 (32). Data supporting the findings of this study cannot be posted publicly due to privacy restrictions. These data are proprietary to the National Academies Press and must be requested from them.
