# Supplementary material for: Widespread use of National Academies consensus reports by the American public
Source: Proc Natl Acad Sci U S A. 2022 Feb 22;119(9):e2107760119. doi: 10.1073/pnas.2107760119 (PMC8892306; doi:10.1073/pnas.2107760119)
Supplement: Supplementary File [file pnas.2107760119.sapp.pdf]

PNAS
www.pnas.org

Supplementary Information for

Widespread use of National Academies consensus reports by the American public

Diana Hicks[a], Matteo Zullo[a,b], Ameet Doshi[a,c], Omar I. Asensio[a,d]

[a] School of Public Policy, Georgia Institute of Technology, Atlanta, GA 30332
[b] Andrew Young School of Policy Studies, Georgia State University, Atlanta, GA 30303
[c] Library, Georgia Institute of Technology, Atlanta, GA 30332
[d] Institute for Data Engineering & Science (IDEaS), Georgia Institute of Technology, Atlanta, GA 30332

Corresponding Author: Diana Hicks
Email: diana.hicks@pubpolicy.gatech.edu

This PDF file includes:

Supplementary text
Tables S1 to S3
SI References

Methods. Records came tagged with a country of origin based on the location of the IP address. This country identification was used to select US downloads and comments. We found several Chinese email providers in the US data, such as @163.com, @126.com, @sina.com, and removed these records. The data include the domain of the downloader and a random number assigned to each user so that users could be counted, but no users could be identified. User numbers allowed algorithmic downloads to be identified and removed, such as 28,000 uncommented downloads over a few hours from a single user using a masking VPN IP address.

There were also 1,559,709 duplicate downloads defined as downloads of the same report on the same day by the same person. We removed these. Comments included people refusing to answer the prompt yet typing in text, for example: ppoo00, nan, kjbkbknln and similar. We excluded 2,051 such examples from the analysis.

Downloads that were commented were similar to uncommented downloads in their geographic and sectoral distributions. Geographic location at the state level was obtained from IP addresses using GeoLite2 data created by MaxMind, available from www.maxmind.com. IP address location is inexact, and a scattering of global locations were pulled in several hundred cases and were excluded. Sectors (see Table 2 in main text) were identified from the domain augmented with manual identification. For example, .edu was identified as universities, .com as companies, .gov as federal government and .org as non-profit. Hospitals, schools, and state and local government were identified through manual identification using keyword searching and pattern matching (hosp for hospital, K-12 for school, for example). The Kolmogorov–Smirnov (K-S) test was used to test that downloads that were and were not commented exhibited equality of distribution across states and across sectors (university, government, .com etc.). The distributions matched closely in both cases. The K-S statistic for distribution across states was 0.12, p-value 0.87. The K-S statistic for distribution across sector was 0.21 with a p-value of 0.92. In both cases, the null hypothesis that the distributions are the same cannot be rejected, and we believe that the distributions are the same. This suggests that the 765,000 people commenting are representative of the 2.4 million people downloading reports.

BERT is a pattern recognition algorithm. Therefore, the data was prepared for processing by improving consistency using a custom Python script. Misspelling is one form of inconsistency in the raw data. Therefore, spelling was corrected. Standard dictionaries were found to introduce more misspellings than they corrected: for example, a common abbreviation of graduate – grad – would be changed to grand. Therefore, a custom misspelling dictionary was created using the Python hunspell package to identify misspellings of the most frequently appearing 20 words in the corpus. In addition, abbreviations associated with NASEM were standardized to NASEM, and other items were added for a total of 1,300 dictionary entries. Inconsistent use of contractions and state abbreviations were regularized through expansion. @ outside an email was replaced with “at”; contentless characters - for example: <, >, slashes and quotes - were removed; multiple ellipses and hyphens were reduced to single characters, and text was transformed to lower case. Finally, personally identifying information, including names, emails, and phone numbers was masked. In total, 70% of comments were edited, and 224k changes were made.

We achieve state-of-the-art topic classification using the transformer-based deep neural network BERT, which stands for bidirectional encoder representations from transformers. Large-scale analysis of unstructured comment data remains challenging, especially when there are many small categories and a few large ones. Such imbalanced data create challenges for previous models (RNN or CNN) to learn important but less frequently occurring labels and often lead to algorithmic bias (1). Even worse were previous unsupervised methods, such as topic modeling, that tended to produce clusters with very little theoretical or social meaning. In this paper, we build on the work of Ha et al. (1) to demonstrate the use of deep neural networks for topic analysis of large free-text comment datasets.

BERT was developed by Google and is now used to process search queries (2, 3). The model was pre-trained by Google on an 11,000 book, 800M word book database built by previous researchers (4) and 2,500M word English Wikipedia. Pre-training involved randomly masking words and training the model to guess the masked word as well as having the model choose the correct 2nd sentence when presented with a first sentence and several options for possible 2nd sentences. These essentially meaningless tasks were a vehicle to train a 12-layer neural network to understand words in context. With the heavy lifting done by Google in the pre-training, BERT users start with a model embodying a great deal of language understanding and train an additional layer specific to their task by feeding the model manually classified examples, called ground truth by computer scientists (5).

We built the ground truth dataset in two stages. In the first, pilot stage, the taxonomy was developed by manual, descriptive coding. The six most frequent comments were: research, education, reference, school, personal use, and teaching. These were classified into the categories: research, learn about, referencing and citation, student, personal, and teaching. At this point, 12% of the comments were classified. Moving on to inspect the top 250 most frequently occurring comments and developing categories to capture their similarities and differences classified one quarter of the comments. Work then shifted to searching the remaining comments for words occurring often in the classified comments, inspecting the results of the keyword searches, and assigning categories to keyword search result sets that were reasonably homogeneous. An iterative process of category refinement, word frequency analysis and keyword search refinement resulted in the final category scheme and classification of 90% of the comments. The appendix table available here: https://doi.org/10.6084/m9.figshare.14605839.v3 lists each category and provides details including the keywords used in the pilot stage to delineate the category, notes on category boundaries, sample comments, as well as most frequently occurring words and phrases.

In the second stage the ground truth training set was constructed. Comments were randomly selected from each of the pilot classification categories. Two researchers inspected each of these comments. Prior research has established that cheaper alternatives, such as using Qualtrics survey respondents, or even trained students, produced inferior results on multi-category classification of sophisticated user comments (1). For a comment to be included in the ground truth set, both researchers had to agree on the category and that only one category applied to the comment. The ground truth set included 28,185 comments, split into a training and validation set of 25,356 comments and a holdout/test set of 2,829 comments used to assess the accuracy of the model's predictions. The equality of comment distribution across states and sectors (university, government, .com etc.) in the ground truth and full dataset was tested using the Kolmogorov–Smirnov (K-S) statistic. The distributions matched closely in both cases. The K-S statistic for distribution across states and sectors were both 0.21, p-value 0.92. In both cases, the null hypothesis that the distributions are the same cannot be rejected, and we believe that the distributions are the same. This suggests that the 28k comments in the ground truth set are a random sample of the full set of 1.6M comments.

We used the FastBert package (6) implementing pytorch-transformers to fine tune the multi-class bert-base-uncased model available through the HuggingFace library (7), and the optimizer used was LAMB (8). Hyperparameter optimization was conducted following Smith (8, 9). The optimal learning rate range was identified by tracking the validation loss progression over a few initial iterations and detecting early overfitting. The optimal learning rate-weight decay combination was chosen using a grid search with learning rate values in the optimal range and standard weight decay values (9). The learning rate increased linearly in the 500 warmup iterations and subsequently diminished following a cosine function. The final hyperparameter specifications were batch size 8, learning rate 1e-3, warmup steps 500, warmup cosine, weight decay 1e-4, training epochs 2 for the 6-category scheme and 3 for the 64-category scheme. The reported standard errors were generated by running 10-fold cross-validation of the optimized model. Importantly, these results are robust to different hyperparameter specifications within the optimal learning rate range. The 64-category prediction was built from the best performing model of a set of 10 runs that averaged 84% (s.e. 0.004) accuracy and F1 macro 0.83 (s.e. 0.005). Table S3 reports category accuracy and F1s. Across the 64 categories, recall averaged 83.2% and precision 83.8%. Training time was 16.7 minutes, computed on 4 NVIDIA Quadro RTX 6000 GPUs.

Baseline comparisons were performed using Naïve Bayes and SVM classifiers. Naïve Bayes achieved an F1 macro of 0.606, and SVM an F1 of 0.735. This establishes that BERT was indeed learning and achieving more than is possible with classical techniques. The pilot keyword classification (for the 87% of records with a keyword classification) had accuracy of 78% and F1 macro of 0.698. As noted, 45% of the comments are duplicates, with the comment "research"

appearing over 100,000 times. The training and testing were performed on a de-duplicated set of comments. If the duplicates are added back into the training and testing set (including "research"), accuracy on the 6-category model becomes 98.4% and F1 97.0, while accuracy on the 64-category model becomes 96.2% and F1 91.2.

We also probed the baseline accuracy of 84%. Although the convention of data sets labeled by humans being called "ground truth" may obscure this point, our classification of comments into 64 categories, indeed the construction of those categories, entailed a degree of arbitrary convention and variability between human classifiers (10). Chi et al. in their assessment of how well BERT learned sentence structure noted the same in relation to annotation conventions of different treebanks and linguistic theories such that BERT's behavior was not clearly wrong (11). To demonstrate this point, three of the authors (AD, DH, MZ) independently reexamined the confusions in the 64 category BERT model, that is the comments on which the ground truth and BERT classifications differed. Re-examiners were presented with each comment along with the ground truth and BERT categories in alphabetical order so that their origin was not known. Re-examiners could choose any of the 64 categories, or none, particularly if they thought that more than one category actually applied to the comment (recall that one criterion for a comment to enter the ground truth set was that in the judgement of the original assessors only one category applied).

After this exercise, we in essence had five votes for the category of each comment. In only one case did all three re-examiners believe that more than one category applied. Table S1 reports the results of this "voting" process. In 58 cases, the three independent judgments agreed with BERT's classification, which we interpret as the original, ground truth, classification of the comment was wrong. In 91 cases the reverse happened, and the three re-examiners agreed with the original ground truth category, so BERT was wrong. In 245 cases, the result was less clear and there was one dissent from agreement with either BERT or ground truth. In 80 cases, confusion triumphed, and a variety of options were offered such that there was no majority which suggests that these comments are unclassifiable in this category scheme.

Table S1 suggests that the 84% accuracy we claim for our classification obscures a more complex picture in which the accuracy might be 85%, if 58 comments were reclassified in a second round prompted by BERT, or 90%, if majority votes were included in that reclassification. It would be unreasonable to say BERT was wrong in an additional 3% of cases where comments are unclassifiable, likely because commenters were ambiguous. Recognizing the limits of humans classifying large numbers of text snippets into complex and subtle category schemes prompts reflection on the strengths BERT brings to the task. BERT's classifying has the virtue of consistency. Whereas people differ in their readings of text and vary in the criteria they apply over the course of a long process, BERT will not. When combined with BERT's high baseline accuracy, this consistency is a virtue that could justify claims of superhuman performance on the classification task.

Table S1. Results of reexamining disagreements between BERT and ground truth comment classifications

**Interpretation**	**Results**	**Number of comments**	**Share of confusions**
Ground truth wrong	3 of 3 agree with BERT	58	12%
BERT wins vote	2 of 3 agree with BERT	126	27%
Unclassifiable	No majority vote	80	17%
Ground truth wins vote	2 of 3 agree with ground truth	119	25%
BERT wrong	3 of 3 agree with ground truth	91	19%
Total		474	100%

Table S2. Explanation of Table S3.

Column	Explanation
Category	Name of category
Accuracy	Percentage of records correctly classified & std error
F1	F1 macro & standard error, measure of machine learning accuracy, harmonic mean of precision and recall
Comments	Number of comments classified into the category
Share	Share of all comments that were classified into the category
Reports	Number of different reports commented upon
Correlation	Correlation coefficient between category count by report and overall count of comments by report
Herfindahl	Herfindahl Index across reports multiplied by 100, a measure of how concentrated are comments on a few reports (higher number) or spread evenly across many reports (a lower number)

Table S3. Category statistics. Table S2 explains each column.

Category	Accuracy	F1	Comments	Share	Reports	Correlation	Herfindahl
research	0.79 (0.014)	0.78 (0.012)	262,812	16.91%	8157	0.80	0.10
student	0.92 (0.017)	0.92 (0.012)	226,287	14.56%	6399	0.77	1.75
teaching	0.77 (0.019)	0.81 (0.012)	158,231	10.18%	5110	0.85	2.21
learn about	0.67 (0.018)	0.72 (0.013)	143,814	9.25%	6863	0.94	0.20
personal	0.87 (0.028)	0.85 (0.020)	122,625	7.89%	6247	0.67	0.18
professional development	0.87 (0.021)	0.85 (0.014)	50,539	3.25%	3936	0.87	1.42
referencing or citation checking	0.67 (0.035)	0.71 (0.018)	47,926	3.08%	5943	0.82	0.11
professional	0.62 (0.022)	0.68 (0.017)	47,319	3.04%	5167	0.74	0.12
teaching administration	0.85 (0.025)	0.79 (0.014)	41,383	2.66%	2318	0.76	5.95
read	0.86 (0.013)	0.87 (0.016)	29,146	1.88%	4331	0.87	0.23
clinical	0.85 (0.016)	0.86 (0.010)	26,146	1.68%	1724	0.37	0.97
transportation	0.96 (0.011)	0.92 (0.011)	25,884	1.67%	2614	0.03	0.33
grant proposal	0.95 (0.013)	0.96 (0.009)	24,162	1.55%	3181	0.61	0.32
sharing	0.88 (0.018)	0.83 (0.013)	21,982	1.41%	2950	0.88	0.35
public health	0.81 (0.026)	0.76 (0.016)	18,034	1.16%	1639	0.22	0.44
policy	0.89 (0.020)	0.86 (0.022)	17,984	1.16%	2992	0.43	0.26
for information	0.83 (0.053)	0.77 (0.045)	16,390	1.05%	3832	0.83	0.13
program design & management	0.67 (0.045)	0.74 (0.030)	16,071	1.03%	3091	0.61	0.19
planning & strategy	0.82 (0.025)	0.88 (0.025)	16,039	1.03%	3070	0.62	0.24
report or presentation	0.95 (0.010)	0.90 (0.013)	15,647	1.01%	3480	0.74	0.24
report review	0.76 (0.020)	0.70 (0.020)	14,312	0.92%	3710	0.86	0.15
committee work	0.90 (0.019)	0.89 (0.013)	12,422	0.80%	2585	0.83	0.34
evaluation	0.80 (0.023)	0.73 (0.019)	10,798	0.69%	2922	0.47	0.22
government	0.73 (0.046)	0.76 (0.041)	10,706	0.69%	3224	0.39	0.09
commercial	0.81 (0.028)	0.76 (0.031)	10,114	0.65%	2857	0.44	0.14
family	0.98 (0.015)	0.98 (0.009)	10,105	0.65%	1566	0.55	0.97
literature review	1.00 (0.025)	0.95 (0.016)	9,464	0.61%	2874	0.49	0.27
media	0.88 (0.028)	0.85 (0.032)	9,097	0.59%	2278	0.59	0.26
res gov	0.88 (0.019)	0.87 (0.016)	9,068	0.58%	1351	0.10	6.10
meeting	0.76 (0.024)	0.80 (0.019)	8,807	0.57%	2240	0.85	1.16
public outreach	0.80 (0.021)	0.85 (0.015)	8,329	0.54%	1776	0.53	0.45
consulting	0.96 (0.009)	0.95 (0.012)	7,669	0.49%	2641	0.65	0.12
NASEM	0.78 (0.033)	0.80 (0.022)	6,894	0.44%	2862	0.61	0.08
environmental work	0.58 (0.030)	0.67 (0.028)	6,440	0.41%	1353	0.09	0.37
gratitude	0.89 (0.000)	0.89 (0.035)	6,397	0.41%	2280	0.87	0.17
book	1.00 (0.016)	0.95 (0.016)	6,359	0.41%	2207	0.83	0.21
law	0.87 (0.026)	0.90 (0.020)	6,322	0.41%	1269	0.10	3.90
improve skills	0.63 (0.026)	0.75 (0.031)	6,320	0.41%	1696	0.81	0.75
veteran	0.93 (0.032)	0.93 (0.015)	5,556	0.36%	491	0.00	3.53
regulatory	0.96 (0.026)	0.94 (0.014)	5,400	0.35%	1536	0.15	0.41
device	0.86 (0.056)	0.80 (0.032)	4,525	0.29%	1764	0.87	0.41
job transition	0.65 (0.064)	0.70 (0.058)	4,434	0.29%	1623	0.84	0.53
serious leisure	0.89 (0.051)	0.87 (0.024)	4,332	0.28%	1435	0.51	0.40
library	1.00 (0.011)	0.97 (0.011)	3,752	0.24%	1704	0.75	0.24
guidelines	0.67 (0.080)	0.70 (0.052)	3,371	0.22%	1347	0.71	1.32

Category	Accuracy	F1	Comments	Share	Reports	Correlation	Herfindahl
helping veterans with VA disability claims	0.82 (0.044)	0.82 (0.051)	3,246	0.21%	390	0.00	2.65
museum	0.80 (0.044)	0.86 (0.033)	3,204	0.21%	679	0.64	3.58
time	0.93 (0.000)	0.97 (0.017)	3,130	0.20%	1373	0.96	0.59
rant	0.80 (0.054)	0.73 (0.037)	2,782	0.18%	1222	0.57	0.34
chronic illness	0.83 (0.047)	0.87 (0.031)	2,605	0.17%	508	0.10	3.56
history	0.91 (0.044)	0.87 (0.029)	2,592	0.17%	1445	0.24	0.16
nonprofit work	0.80 (0.057)	0.84 (0.032)	2,505	0.16%	1064	0.54	0.32
retired	1.00 (0.000)	0.93 (0.026)	2,312	0.15%	1001	0.54	0.48
uncertain	1.00 (0.047)	1.00 (0.027)	2,188	0.14%	1151	0.80	0.24
debate and competition	0.80 (0.032)	0.89 (0.019)	1,902	0.12%	689	0.22	1.00
farm	1.00 (0.092)	0.89 (0.060)	1,569	0.10%	327	0.02	5.83
dwnld for another	0.85 (0.036)	0.76 (0.034)	1,288	0.08%	741	0.92	0.45
delight	0.33 (0.176)	0.50 (0.139)	1,196	0.08%	812	0.83	0.26
journal/book club	0.25 (0.088)	0.36 (0.105)	1,186	0.08%	406	0.64	1.67
homeschool	1.00 (0.000)	0.86 (0.083)	860	0.06%	255	0.75	6.07
fiction writers	0.60 (0.140)	0.75 (0.106)	698	0.04%	455	0.35	0.47
frustration	1.00 (0.225)	0.86 (0.149)	605	0.04%	448	0.84	0.44
accreditation	1.00 (0.000)	1.00 (0.000)	449	0.03%	153	0.07	8.43
church work	1.00 (0.000)	1.00 (0.137)	428	0.03%	178	0.17	11.88

SI References

1. S. Ha, D. J. Marchetto, S. Dharur, O. Asensio, Topic classification of electric vehicle consumer experiences with transformer-based deep learning. *Patterns* **2**, 100195 (2021).
2. J. Devlin, M. W. Chang, K. Lee, K. Toutanova, Bert: Pre-training of deep bidirectional transformers for language understanding. arXiv [Preprint] (2018). https://arxiv.org/abs/1810.04805v2 (accessed 7 May 2021).
3. A. Vaswani, et al., "Attention is all you need" in *Proceedings of the 31st International Conference on Neural Information Processing Systems*, (2017). pp. 6000-6010.
4. Y. Zhu, et al., "Aligning books and movies: Towards story-like visual explanations by watching movies and reading books" in *Proceedings of the IEEE international conference on computer vision*, (2015). pp. 19-27.
5. H. Xu, B. Liu, L. Shu, P. Yu, "BERT Post-Training for Review Reading Comprehension and Aspect-based Sentiment Analysis" in *Proceedings of the 2019 Conference of the North American Chapter of the Association for Computational Linguistics: Human Language Technologies,* (2019). (Vol. 1).
6. K. Trivedi, FastBert. GitHub. http://github.com/kaushaltrivedi/fast-bert. (Deposited 17 May 2019).
7. T. Wolf, et al., HuggingFace's Transformers: State-of-the-Art Natural Language Processing. arXiv [Preprint] (2019). http://arxiv.org/abs/1910.03771 (accessed 7 May 2021).
8. L. N. Smith, "Cyclical learning rates for training neural networks" in *2017 IEEE winter conference on applications of computer vision (WACV)*, (2017). pp. 464-472.
9. L. N. Smith, A disciplined approach to neural network hyper-parameters: Part 1--learning rate, batch size, momentum, and weight decay. arXiv [Preprint] (2018). https://arxiv.org/abs/1803.09820v2 (accessed 7 May 2021).
10. L. Aroyo, C. Welty. Truth is a lie: Crowd truth and the seven myths of human annotation. *AI Magazine* 36, 1:15-24 (2015).
11. E. A. Chi, J. Hewitt, C. D. Manning, "Finding Universal Grammatical Relations in Multilingual BERT" in *Proceedings of the 58th Annual Meeting of the Association for Computational Linguistics*, (2020). pp. 5564-5577.
